# Modelling the Associations Between the Physician–Patient Relationship and Patient Outcomes via Self-Determination Theory Variables in Chronic Disease Management

**DOI:** 10.1007/s12529-025-10371-0

**Published:** 2025-06-27

**Authors:** Jessica A. Eigeland, Robyn L. Moffitt, Nicola Sheeran, Natalie Loxton, Liz Jones

**Affiliations:** 1 School of Applied Psychology, Mt Gravatt Campus, Griffith University, 176 Messines Ridge Road, Mount Gravatt 4122, Brisbane, QLD Australia; 2 School of Psychology, Faculty of Health, Deakin University, 1 Gheringhap Stress, Geelong, VIC 3220 Australia; 3 Department of Psychology, Monash University Malaysia, Kuala Lumpur, Malaysia; 4 Department of Psychology, Monash University, Selangor Darul Ehsan, Jalan Lagoon Selatan, 47500 Bandar Sunway, Malaysia

**Keywords:** Physician-patient relationship, Chronic conditions, Self-determination, Autonomous motivation, Adherence

## Abstract

**Background:**

The aim of this study was to model the associations between the physician–patient relationship and patient outcomes, considering the mediating role of Self-Determination Theory variables.

**Method:**

The current study comprised a sample (*N* = 226) of Australians with a diagnosed chronic health condition. Path analysis via structural equation modelling was used to model the relationships between patients’ self-reported perception of the physician–patient relationship, motivation (autonomous and controlled), basic psychological needs (satisfaction and frustration), and patient outcomes (treatment adherence, mental health, and physical health).

**Results:**

Stronger physician–patient relationships indirectly predicted better physical and mental health, as well as greater adherence to treatment. This occurred through serial mediation, whereby stronger physician–patient relationships directly predicted need satisfaction, which in turn predicted autonomous motivation. Conversely, weaker physician–patient relationships indirectly predicted poorer mental health and lower adherence to treatment. This occurred through serial mediation where weaker physician–patient relationships predicted need frustration, which in turn predicted controlled motivation. Weaker physician–patient relationships also directly predicted controlled motivation.

**Conclusion:**

Satisfaction of basic psychological needs plays an important role in predicting patient motivation and outcomes. The current findings provide evidence that the physician–patient relationship plays a direct role in facilitating satisfaction and frustration of psychological needs, as well as patient motivation. Attention should be placed on interventions aimed at improving the physician–patient relationship as a means through which to increase need satisfaction, autonomous motivation, and, consequently, patient adherence and outcomes.

## Introduction

Globally, the prevalence of chronic diseases in developed countries is increasing among all age, sex, social economic status (SES), and ethnic groups [1, 2]. Within Australia, survey data from 2020 to 2021 revealed that 47% of Australians had at least one chronic disease and another 20% had two or more, with 89% of deaths in 2020 being associated with chronic diseases [1]. Moreover, chronic diseases significantly contribute to substantial burden for individuals and society, due to their long-lasting effect on physical and mental health, frequent healthcare utilisation, loss of productivity, and reduced health-related quality of life [1, 3, 4]. In conjunction, rates of depression (60%), anxiety (61%), and stress (49%) among those with a chronic disease are also higher than prevalence rates in the general population [5, 6]. While most chronic health conditions cannot be cured, their symptoms can usually be managed with behavioural changes including eating a healthy diet, engaging in regular physical exercise, abstaining from tobacco, and taking recommended medications. These behavioural changes require active engagement, self-monitoring, and maintenance by the patient. Recently, rates of medication non-adherence among chronic disease patients have been reported at 45.5% [7] with low adherence (i.e. not strictly adhering to physician recommendations) reported to be as high as 85% [8]. Poor health-related quality of life is also commonly reported, particularly for patients with lower adherence behaviours [9, 10]. Thus, understanding what motivates the uptake and maintenance of recommended health behaviours is of importance for patient health and well-being.

Reasons for non-adherence to treatment recommendations include income, age, access to treatments, health literacy, and the quality of the physician–patient relationship [11–15]. Moreover, several studies indicate that the quality of the physician–patient relationship significantly impacts patients’ decision-making regarding adherence to treatment [16–20]. For example, Mahmoudian, Zamani [18] found that lower satisfaction within the physician–patient relationship was associated with non-adherence to medication after controlling for the physicians’ gender and the patients’ age, gender, education, and duration of disease. Similarly, Orom, Underwood [21] found that patients who reported a stronger physician–patient relationship were more likely to follow the recommended treatment plan. Subsequently, a large body of research has focused on improving the quality of the physician–patient relationship as a mechanism through which to improve treatment adherence and patient outcomes [18, 22–25]. This research has typically focused on improving physician communication skills with respect to relational elements including empathy, trust, liking, and respect [26–28]. Research has also demonstrated the importance of communication skills that foster and support patient empowerment, self-efficacy, and autonomy for improving the physician–patient relationship and patient outcomes [29–34]. However, there is a scarcity of research utilising motivational theory to understand the psychological processes through which the physician–patient relationship may relate to adherence and patient outcomes in individuals with chronic conditions.

The self-determination theory (SDT) [35, 36] has been applied to the study of behaviour change in a wide range of domains, including health [37, 38]. According to SDT, the initiation and maintenance of behaviours over time requires that patients internalise values and skills for change and experience self-determination. This is particularly important for patients with chronic diseases, where self-management plays a critical role in ongoing care and overall well-being [39]. One of the central themes of SDT is the distinction between two forms of self-regulation, “autonomous motivation” (i.e. engaging in a behaviour because it is consistent with intrinsic goals or values and emanates from the self) and “controlled motivation” (i.e. engaging in behaviours for externally referenced reasons, such as to gain rewards or perceived approval from others or to avoid punishment or feelings of guilt). Research consistently demonstrates that autonomous forms of motivation are more beneficial than controlled forms of motivation for long-term maintenance of health behaviours [40, 41]. Further, the theory suggests that the satisfaction of three key psychological needs, autonomy (feeling a sense of choice about one’s treatment decisions), competence (having self-confidence and self-efficacy regarding what behaviours are required to change), and relatedness (feeling a sense of being close to, and understood by, their physician) can facilitate more autonomous motivation and, consequently, long-term maintenance of health behaviours [38, 42–44].

In the chronic disease context, research utilising SDT has consistently reported a positive association between the satisfaction of psychological needs and autonomous motivation [38, 45–47]. Autonomous motivation has subsequently been associated with patients being more likely to engage in self-management behaviours promoted by physicians and report better physical and mental health outcomes [40, 45, 48, 49]. For example, Williams, McGregor [50] found that higher autonomous motivation predicted greater improvement in glycaemic control at both 6- and 12-months follow-up in a randomised trial of patients with type 2 diabetes. Conversely, according to SDT theory, thwarting or frustration of these basic psychological needs can undermine self-determined motivation [36]. Similarly, controlled motivation can perpetuate maladaptive coping mechanisms and has been linked with greater alcohol use, smoking, and binge eating behaviours [51–54]. For instance, severely obese individuals involved in a dieting programme who reported controlled reasons for change lost less weight, were more likely to quit earlier, and demonstrated poorer maintenance behaviours. More recently, frustration of basic psychological needs was found to be related to lower usage of self-monitoring devices in a sample of primary care patients [42]. The relationship between basic psychological needs and the physician–patient relationship has been scarcely studied. Understanding how to utilise the physician–patient relationship to increase satisfaction of basic psychological needs may aid in patient adherence to treatment and improved health outcomes.

Research suggests that satisfaction of patient’s basic psychological needs is influenced by the interaction style of the physician, alongside personality differences between patients, and the intrinsic or extrinsic nature of the patient’s health-related goals [55]. Similarly, research has demonstrated that patients who perceive their physicians as autonomy supportive also report greater need satisfaction and higher healthcare indices such as quality of life, mental health, and adherence to treatment [43, 48, 50, 56–58]. The concept of autonomy support applied to health contexts describes an interpersonal orientation in which the physicians consider the perspectives and experiences of the patient, provide opportunities for patient choice of treatment, and encourage active involvement in treatment-related decision-making [59]. Support for these basic psychological needs is also afforded when physicians provide relevant information and rationale for change, while also supporting patients as they explore their own personal resistance and barriers to change [59, 60]. By contrast, physicians who motivate patients through incentives or contingent approval may elicit more controlled forms of motivation within patients and, consequently, poorer health outcomes [61, 62].

Studies have also demonstrated that physicians can learn to be more autonomy supportive by adopting SDT-based interaction styles [56, 63]. SDT-informed physician–patient interactions are “patient-centred” because they encourage physicians to seek to understand and utilise the patient’s perspective and experiences while supporting patient autonomy [64]. This patient-centred approach was first identified in the 1950 s and has since been considered an important element in the establishment of the physician–patient relationship [65]. Empirical research from the communication literature has demonstrated that specific physician behaviours during the medical interview such as eliciting patients’ perspective and opinions, not interrupting patients, use of open and closed questions allowing patients to describe their experiences, and use of summaries were all correlated with higher patient disclosure of information required for accurate diagnosis. These findings remained consistent after controlling for consultation time [66]. Similarly, recent research has highlighted that patients prefer physicians who “take on board my suggestions and opinions regarding my health”, “validate my experience”, and “ask how things in my life impact my ability to manage my illness and try to assist me with this as much as possible” in order to establish a good physician–patient relationship [67]. Such behaviours are consistent with support for patient needs for autonomy, relatedness, and competence.

### The Present Study

Although the importance of physician–patient interactions that are patient-centred and autonomy supportive is well established, as is the relationship between psychological needs, self-regulation, and health outcomes, it remains unexplored how the quality of the physician–patient relationship directly relates to these SDT variables, and, in turn, patient outcomes. Understanding the role that SDT variables play in the link between the physician–patient relationship and patient outcomes may help tailor interventions to improve patient motivation to engage in healthcare behaviours and ultimately contribute to improving patient outcomes. Accordingly, the current study examined how the physician–patient relationship and SDT variables interrelate to predict patient outcomes for those with a chronic health condition.

**Fig. 1:**
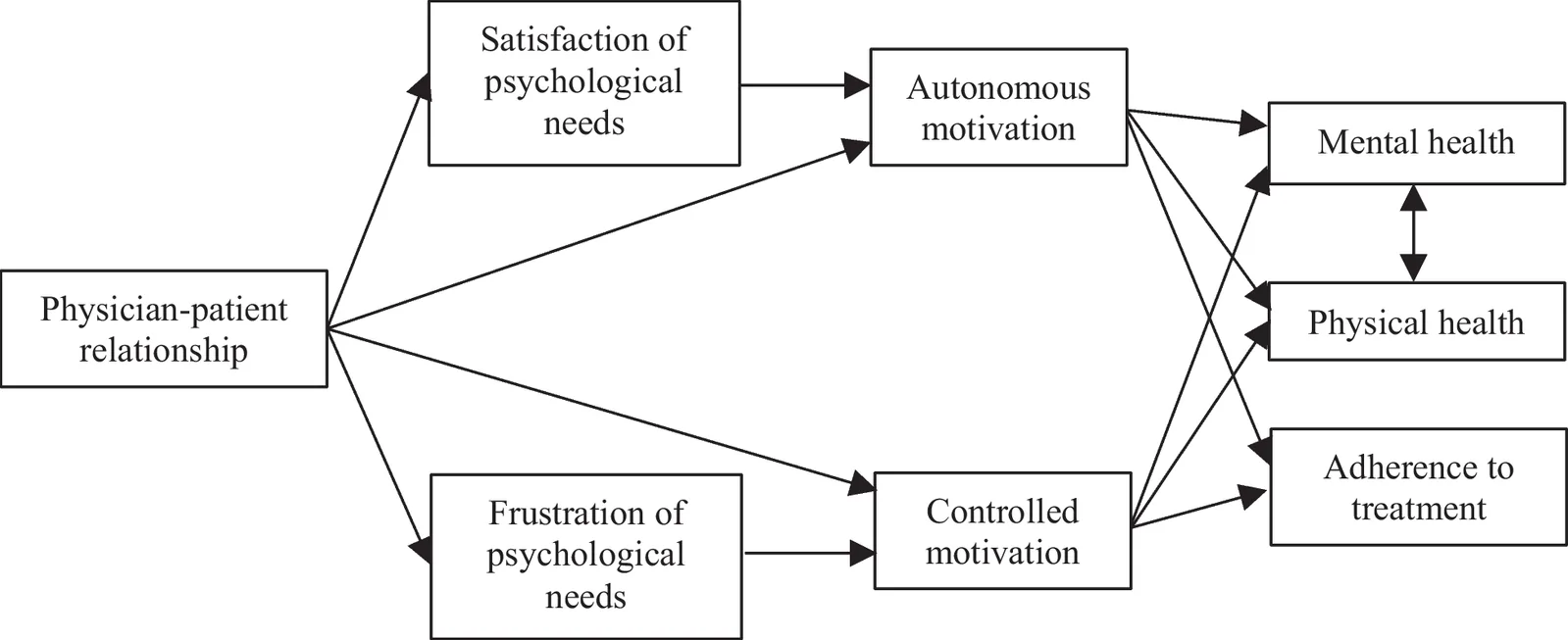
Hypothesised model (model 1) for the relationship between the physician–patient relationship, SDT variables, and patient outcomes

Informed by previous research, we developed and tested a new path model displayed in Fig. 1.

## Methods

### Procedure

Ethical clearance was obtained from Griffith University Human Ethics Committee (HREC number 2019/982). Recruitment occurred through social media, e-newsletters distributed by large health organisations, and via emails to staff, students, and alumni of the university. Recruitment occurred for approximately 12 months (2022–2023) until a sufficient sample size was achieved. Participants were eligible to participate if they answered yes to a series of screening questions. These were the following: (a) currently residing in Australia, (b) 18 years of age or older, (c) had a chronic health condition excluding mental health conditions, conditions which significantly impact memory (e.g. Alzheimer’s), and life-limiting conditions (e.g. cancer), (d) were proficient in English, and (e) had seen their physician within the last 12 months. The latter allowed for accurate recall of physician behaviours relevant to the present study. Eligible participants were directed to an online survey (hosted on Lime Survey), which contained a participant information sheet, consent form, and questions about the physician the participant felt primarily treated their chronic health condition. As an incentive to participate, participants could enter a prize draw to win one of three $100 gift vouchers.

### Measures

#### Physician–Patient Relationship

The 22-item Chronic Condition Physician–Patient Relationship Scale (CC-PPR) was used to assess the frequency of physician behaviours critical to the formation of a good physician–patient relationship in patients with chronic physical health conditions [68]. Participants were asked to rate how often the physician who primarily treated their chronic physical health condition displayed each of the 22 behavioural items on a 5-point Likert scale from “Never = 1” to “Always = 5”. Scores were averaged, with higher scores indicating a stronger physician–patient relationship. The CC-PPR has shown excellent internal validity (Cronbach’s α = 0.97). In the current study, Cronbach’s alpha was found to be 0.98.

#### Measure of Psychological Needs

To assess need satisfaction and frustration, we adapted the Balanced Measure of Psychological Needs [69]. Satisfaction of basic psychological needs was measured using nine items (3 items assessing autonomy, 3 assessing competence, and 3 assessing relatedness). A further nine items were used to measure frustration of basic psychological needs (again with 3 items for autonomy, 3 for competence, and 3 for relatedness). Examples of items included: “When I am with my doctor, I have a say in what happens, and I can voice my opinion”, “When I am with my doctor, I often feel inadequate or incompetent.”, and “When I am with my doctor, I feel cared about”. Items were rated on a 7-point Likert scale, ranging from “Not at all true = 1” to “Very true = 7”. Scores were averaged for both scales separately, with higher scores indicating a higher need satisfaction or frustration, respectively. In the current study, Cronbach’s alpha for the satisfaction and frustration scales was 0.93 and 0.89, respectively.

#### Motivation

The 12-item Treatment Self-Regulation Questionnaires (TSRQ; Levesque et al., 2007) were used to assess the degree to which a person’s motivation for particular health behaviours was autonomously regulated versus controlled. There were 6 items assessing autonomous motivation and 6 assessing controlled motivation. Participants were presented with the item stem: “The reasons I would follow my physician’s treatment advice is…”. Examples of controlled reasons included the following: “Because I feel pressure from others to do so” and “Because I want others to see I can do it.” Examples of autonomous reasons included the following: “Because I want to take responsibility for my own health” and “Because I personally believe it is the best thing for my health.” Participants rated each reason on a 7-point Likert scale, ranging from “Not at all true = 1” to “Very true = 7”. Higher scores indicated a greater level of controlled or autonomous motivation, respectively. In the current study, Cronbach’s alpha for the autonomous and controlled subscales was 0.90 and 0.82, respectively.

#### Mental Health

Mental health was measured using the Depression Anxiety and Stress Scale-21 (DASS-21) [70]. Participants were asked to think about their experiences in the past seven days and to judge how each statement applied to them. Each of the 21 items was answered on a 4-point Likert scale ranging from 0 “Did not apply to me at all–Never” to 3 “Applied to me very much, or most of the time–Almost always”. For the current study, a total score was calculated by averaging the 21 items, with higher scores representing poorer mental health. Within the current study, Cronbach’s alpha was 0.95.

#### Physical Health

Physical health was assessed via the 6-item physical health component (PCS) subscale of the Short-Form Health Survey (Ware et al., 1996). The SF-12 is a self-report generic assessment of a patient’s health-related quality of life and has been validated in both the general population and for a range of medical conditions (Gandek et al., 1998; Sanderson & Andrews, 2002). The PCS items measured the patient’s physical function, role limitations caused by physical problems, bodily pain, and general health. A total score was calculated, as per Ware et al. (1998), and then normed to Australian samples using Australian population health data provided by Tucker et al. (2010). Higher scores on PCS represented better physical health. In the current study, Cronbach’s alpha for the PCS was 0.82.

#### Adherence to Treatment

Adherence to treatment was measured using the 5-item General Adherence Scale [71]. Questions (i.e. “I found it easy to do the things my doctor suggested I do”) were responded to on a 5-point Likert scale ranging from 0 “none of the time” to 4 “all of the time”. Scores were calculated by averaging responses to the five items and then transforming the result linearly to a 0–100 distribution. Higher scores reflected greater adherence to treatment. Cronbach’s alpha in the current study was 0.85.

### Data Analysis

Path analysis was used to test the proposed model using AMOS 29 statistical software, with modification indices used to improve fit [72]. Model fit was evaluated against the chi-square (*χ*^2^) goodness of fit index statistic, Tucker-Lewis Index (TLI), Comparative Fit Index (CFI), root mean square error of approximation (RMSEA), and the standardised root mean square residual (SRMR). For acceptable fit, the normed *χ*^2^ should be between 1 and 3, TLI and CFI should be ≥ 0.90, RMSEA should be ≤ 0.06, and SRMR should be ≤ 0.08 [73].

## Results

A total of 226 individuals completed all questionnaires in the study, comprising 176 females, 44 males, and 6 participants who identified as non-binary. The sample ranged in age from 18 to 80 years, with a mean age of 39.34 years (*SD* = 16.69 years). The ethnic composition of the sample was 84% Caucasian/White, 8.6% Asian, 0.9% Aboriginal or Torres Strait Islander, Middle Eastern, Pacific Islander, or Hispanic. The remaining 3.8% identified as other or preferred not to answer. The sample was educated, with 71.8% having at least some level of tertiary education. The mean household income was $79,208 (AUD). A range of chronic health conditions were reported, with musculoskeletal (16.4%), respiratory (12.8%), pain/fatigue (12.4%), endocrine (11.1%), gastrological (8.8%), and gynaecological (8.4%) conditions being the most prevalent. Most participants (80.2%) reported requiring ongoing medication. Participants reported seeing a physician (54.9% male physician; 45.1% female physician) specialising in general practice (78.7%), endocrinology (7.5%), rheumatology (4.9%), pain (4.0%), cardiology (1.3%), immunology (1.7%), or other (2.3%). The majority (54.4%) of participants reported spending between 15 to 30 min with their physician for each consultation. See supplementary Table for further sample characteristics.

IBM/SPSS 28.0 software was used to examine missing data and provide descriptive statistics, alphas, and intercorrelations for all study variables. The dataset contained 1.53% missing data overall, with 11.06% of participants having missing data on 33.33% of variables. Variables with missing data had between 0.4% and 6.6% missing data. Little’s MCAR test for all variables was not significant (*χ*^2^ = 90.24, *df* = 82,* p* = 0.25), indicating data was missing completely at random. Missing data were replaced using the Expectation–Maximisation (EM) technique to maximise sample size for analysis.

Table 1 presents means, *SD*s, and intercorrelations for all variables assessed in this study. Most correlations were statistically significant in the predicted direction, except for the correlation between controlled motivation and physical health, which (while in the predicted direction) was extremely weak (*r* = − 0.03) and non-significant (*p* > 0.05). This path was subsequently trimmed from the model.

**Table 1:** Descriptive statistics, alphas, and intercorrelations for predictor and outcome variables

Variable	*M*	*SD*	Range	1	2	3	4	5	6	7
1. Physician–patient relationship	2.69	1.06	0–4	-						
2. Satisfaction of psychological needs	3.89	1.36	0–6	.85**	-					
3. Frustration of psychological needs	1.53	1.17	0–6	−.77**	−.78**	-				
4. Autonomous motivation	4.91	1.10	0–6	.51**	.57**	−.53**	-			
5. Controlled motivation	2.58	1.46	0–6	−.03	−.02	.23**	−.01	-		
6. Mental Health	6.41	4.27	0–21	−.20**	−.27**	.35**	−.26**	.20**	-	
7. Physical Health	49.86	9.95	0–100	.10	.12	−.16*	.13*	−.03	−.38**	-
8. Adherence to treatment	59.77	14.30	0–100	.34**	.41**	−.38**	.49**	−.19**	−.31**	.16*

** *p* <.001, *p <.05. Range represents possible ranges from scales, not actual range in dataset

### Path Analysis and Tests of Direct and Indirect Effects

**Fig. 2:**
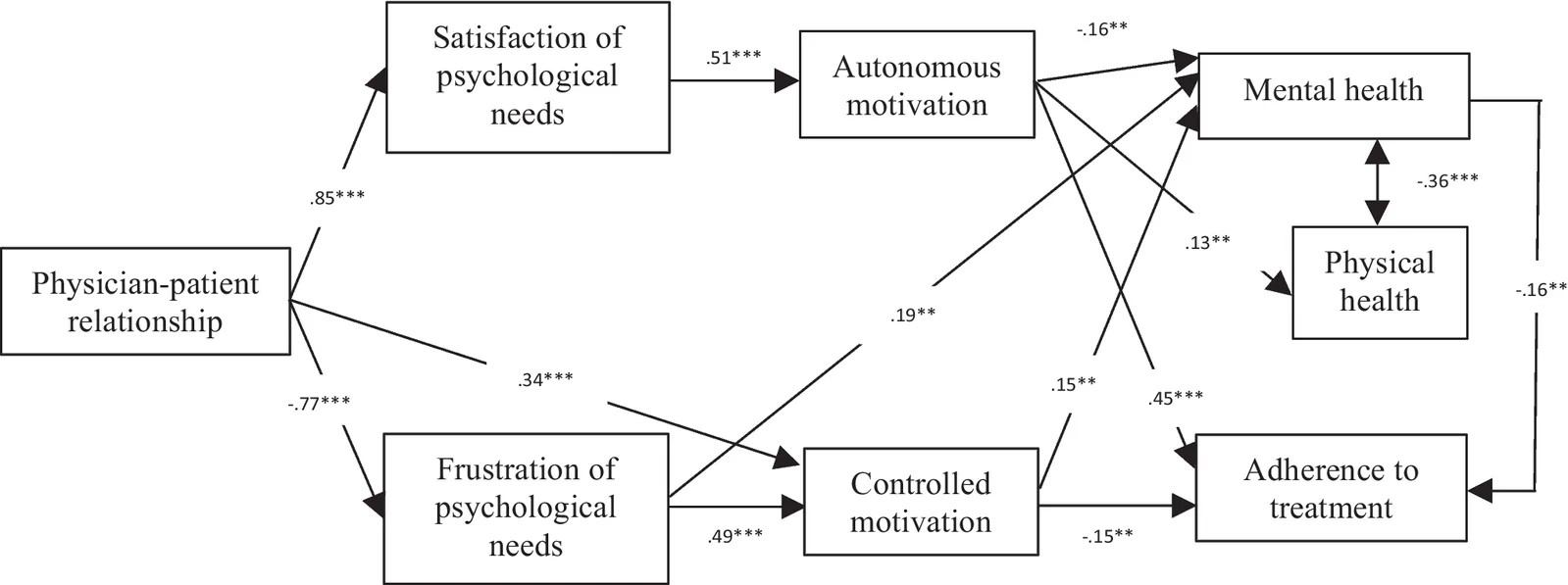
Final model for the relationship between the physician–patient relationship, SDT variables, and patient outcomes (**p <.05, ***p <.001)

As shown in Table 2, the initial proposed model (Model 1) showed some evidence of misfit (RMSEA = 0.08). Modification indices suggested allowing the error covariances between mental health and treatment adherence to be correlated (Model 2) and to free the path from frustration of basic psychological needs to mental health (Model 3). Freeing these paths was considered justifiable given the literature indicating satisfaction of basic psychological needs is associated with more positive affect, less negative affect, and fewer symptoms of poor mental health [35, 74]. Similarly, research has revealed an association between mental well-being and adherence in a range of chronic health conditions [75]. Freeing each parameter resulted in a significant change in chi-square. Model 3 demonstrated good model fit across all indices of fit (normed *c*^2^ = 1.77, CFI = 0.99, TLI = 0.97, RMSEA = 0.06, SRMR = 0.04). Subsequently, Model 3 was deemed the best fitting model for the data. Statistically significant (*p* < 0.05) standardised path coefficients for Model 3 are presented in Fig. 2. Results for tests of indirect effects are presented in Table 3.

**Table 2:** Model fit comparisons

Model	*χ*^2^	*df*	*χ*^2^ change	Normed *χ*^2^	TLI	CFI	RMSEA	SRMR
Model 1: Hypothesised model	37.80***	15	-	2.52	.95	.97	.08	.06
Model 2: Model 1 plus additional path from Adherence to Mental Health	30.21***	14	7.68***	2.16	.96	.98	.07	.05
Model 3: Model 2 plus additional path from Dissatisfaction of psychological needs to Mental Health	23.07**	13	7.14***	1.77	.97	.99	.06	.04

***p <.001, **p <.05

**Table 3:** Indirect effects statistics from model 3

Path	Unstandardised estimates	Lower 95 percent CI	Upper 95 percent CI
PPR–SBPN–AM	.45**	.29	.69
PPR–SBPN–AM–MH	−.27**	−.70	−.06
PPR–SBPN–AM–PH	.53	−.06	1.08
PPR–SBPN–AM–ATT	2.63**	1.45	4.27
PPR–SBPN–AM–MH–ATT	.15**	.03	.58
PPR–DBPN–CM	−.51**	−.70	−.32
PPR–DBPN–MH	−.59**	−.91	−.06
PPR–DBPN–MH–ATT	−.18**	−.46	−.18
PPR–CM–MH	.20**	.01	.45
PPR–CM–MH–ATT	−.11**	−.34	−.01
PPR–CM–ATT	−.68**	− 1.54	−.20
PPR–DBPN–CM–MH	−.22**	−.48	−.02
PPR–DBPN–CM–MH–ATT	.12**	.01	.31
PPR–DBPN–CM–ATT	.75**	.26	1.65

*PPR* physician–patient relationship, *SBPN* satisfaction of basic psychological needs, *DBPN* dissatisfaction of basic psychological needs, *MH* mental health, *PH* physical health, *AT* adherence to treatment, *AM* autonomous motivation, *CM* controlled motivation

***p <.001, **p <.05

The physician–patient relationship showed a significant positive effect on need satisfaction and a significant negative effect on need frustration. Additionally, the physician–patient relationship had a direct positive effect on controlled motivation and an indirect effect on controlled motivation through need frustration. Contrary to expectations, results indicated there was no direct relationship between the physician–patient relationship and autonomous motivation; however, an indirect effect was demonstrated through need satisfaction. Results indicate that stronger physician–patient relationships are associated with more autonomous motivation and decreased controlled motivation, with these relationships being mediated through satisfaction and frustration of basic psychological needs, respectively.

Autonomous motivation in turn had a significant direct effect on adherence to treatment, physical health, and mental health, with greater levels of autonomous motivation being associated with increased adherence to treatment and better physical and mental health. Likewise, controlled motivation had a significant direct effect on mental health and adherence to treatment, indicating that increased levels of controlled motivation were associated with poorer mental health and less adherence to treatment. Need frustration was also directly associated with poorer mental health, and mental health was directly associated with poorer physical health and less adherence to treatment.

The physician–patient relationship was shown to predict mental health and adherence to treatment via all indirect paths shown in Table 3. Specifically, a stronger physician–patient relationship predicted better patient mental health and adherence to treatment through the serial mediation of increased satisfaction of basic psychological needs and autonomous motivation. A stronger physician–patient relationship also predicted adherence to treatment through the serial mediation involving satisfaction of basic psychological needs, autonomous motivation, and better patient mental health. Conversely, weaker physician–patient relationships were shown to predict worse adherence to treatment and poorer mental health through the serial mediation of frustration of basic psychological needs and controlled motivation. Weaker physician–patient relationships also indirectly predicted poorer mental health and worse adherence to treatment through controlled motivation alone. Unexpectedly, there was no direct or indirect relationship between the physician–patient relationship and physical health. The only predictor of physical health found in the current model was autonomous motivation and patient mental health.

## Discussion

The aim of this study was to model the relationships between the physician–patient relationship and patient outcomes considering the SDT variables of basic psychological needs (frustration and satisfaction) and motivation (controlled and autonomous) as mediators. Outcome variables included mental health, physical health, and adherence to treatment. Overall, the findings suggest that the patient’s self-reported quality of the physician–patient relationship can influence satisfaction or frustration of psychological needs, which can, in turn, influence patient motivational orientation and patient outcomes. Consistent with SDT, controlled motivation predicted worse outcomes, whereas autonomous motivation predicted better patient outcomes.

Contrary to expectations, there was no direct relationship between the physician–patient relationship and autonomous motivation. Rather, satisfaction of basic psychological needs mediated the association between these variables. Previous research highlighting the role of the physician–patient relationship on patient motivation has not evaluated the mediating role of basic psychological needs in this relationship [29–34]. In hindsight, the way in which the physician–patient relationship was measured in this study as outlined by Eigeland, Sheeran [68] encompasses themes associated with autonomy, competence, and relatedness. Consequently, in this study, stronger physician–patient relationships correlated strongly with satisfaction of basic psychological needs, so much so that satisfaction of basic psychological needs entirety mediated the relationship between the physician–patient relationship and autonomous motivation. While autonomy-supportive consultations have previously been shown to facilitate a patient’s autonomous decision-making [76], this appears to occur via satisfaction of a patient’s basic psychological needs.

In addition, we hypothesised a direct relationship between controlled motivation and physical health. However, results instead have indicated these variables were indirectly related through mental health. Again, the research suggesting a direct relationship between controlled motivation and physical health did not account for the mediating role of mental health [61, 62]. Controlled motivation, which involves external pressures or rewards to engage in an activity, may not be consistently directly associated with physical health. This may be due to the more direct relationship that mental health has on behaviour change (i.e. following treatment advise) in this population. Mental health conditions like depression and anxiety can significantly impact an individual’s ability to adhere to treatment plans and engage in self-care behaviours. This can lead to difficulties in managing symptoms, adhering to medication regimens, and maintaining a healthy lifestyle [77, 78].

### Theoretical Implications

This study is the first to investigate the relationships between the physician–patient relationship, satisfaction, and frustration of basic psychological needs (i.e. the need for autonomy, competence and relatedness), motivation, and patient outcomes. The present study has provided novel evidence that the strength of the physician–patient relationship directly predicted satisfaction and frustration of basic psychological needs, which in turn predicted motivation orientation, patient adherence, and mental health. Specifically, our findings have demonstrated a need for physician interaction styles that specifically target satisfaction of a patient’s need for autonomy, competence, and relatedness to allow for autonomous motivation and improved health outcomes. Although autonomy and competence have previously been linked with patient adherence and health-related outcomes [37, 79, 80], the role of the physician–patient relationship in these associations, as well as the importance of the psychological need of relatedness, had previously received little consideration.

The current study has also been the first to delineate the link between basic psychological needs, motivations, and patient outcomes. Satisfaction of basic psychological needs mediated the relationship between the physician–patient relationship and autonomous motivation. Frustration of basic psychological needs mediated the role between the physician–patient relationship and controlled motivation. Autonomous motivation, in turn, predicted better outcomes across all indices, while controlled motivation predicted poorer mental health and worse adherence to treatment. Additionally, the current study has provided evidence suggesting that weaker physician–patient relationships were associated with controlled motivation and, indirectly, predicted poorer mental health and worse adherence to treatment. These findings, in combination, add to our theoretical understanding of chronic illness patients’ motivation to engage in health behaviours, suggesting that need satisfaction is an important predictor of more intrinsically oriented, and thus more sustained, motivation. The theoretical model proposed here highlights the mechanisms through which the physician–patient relationship can indirectly influence chronic illness patient outcomes. This is the first known model to report these associations to date, and it may assist physicians and policy makers to tailor interventions to improve patient motivation to engage in healthcare behaviours and, ultimately, to improve patient physical and mental health outcomes.

### Practical Implications

For practitioners, these findings suggest that motivating patients to engage in health behaviours should involve attempts to foster autonomous forms of motivation. This is best facilitated by striving to support patient autonomy, assisting patients to feel a sense of competence to maintain health behaviours, and fostering a sense of relational connection between physician and patient. Previous research has provided concrete behaviours that physicians can use to achieve such goals [67]. Items such as “My doctor will take on board my suggestions and opinions regarding my health”, “My doctor individualizes treatment plans to suit my lifestyle and what I think I can achieve”, and “My doctor validates my experience (e.g. says validating things like “what you’re experiencing is real and valid, and concerning”)” speak to aspects of autonomy, competence, and relatedness. Allocating time in consultations to satisfy all three psychological needs may prove beneficial in fostering autonomous motivation and, consequently, assisting patients to initiate and maintain health behaviours, adhere to treatment regimes, and feel better mentally and physically [41]. In contrast, aiming to motivate patients through external rewards, punishments, or social pressures to receive approval or praise, or to avoid disapproval from others should be avoided due to the relationship between these more controlled forms of motivation and poorer adherence and mental illness.

The current study also highlighted the direct relationship between patient mental illness and adherence to treatment. Rates of depression and anxiety in populations of those with chronic conditions remain high [5, 6, 78]. It, therefore, remains important to consider mental health as an important patient outcome, but also as a potential mechanism through which to positively influence adherence to treatment. Routine monitoring of mental health, alongside discussions of how a patient’s mental health may be impacting their ability to initiate and maintain health behaviours, may prove beneficial. Frustration of basic psychological needs was also directly associated with poorer mental health and indirectly associated with lower adherence to treatment. According to SDT, satisfaction of psychological needs is universally important for psychological well-being [35]. It is perhaps not surprising that the results from the present study indicated that frustration of these needs predicted poorer mental health.

The current findings may assist in the design of intervention programmes aimed at improving patient motivation and adherence to treatment. Studies have shown that autonomous motivation and satisfaction of basic psychological needs are context specific and can be increased through intervention [37, 41, 81]. However, it is important to further delineate which specific techniques or strategies can lead to improvements in patient motivation and satisfaction of psychological needs, especially in the context of the self-management of chronic disease, and this is an important avenue for further research. The current study has provided concrete physician behaviours that have been shown to relate to both autonomous motivation and satisfaction of basic psychological needs, and these behaviours may be a useful starting point to inform such investigations.

### Limitations and Future Directions

Our study has several limitations. Firstly, there was significant gender disparity within our sample, with more female participants and more male physicians. We also recognise that patients’ perspectives can be influenced by a range of factors, including self-awareness [82], illness severity, time since diagnosis, length of the physician–patient relationship, communication preferences, health literacy, and cultural and social norms [83–85]. The generalisability of our findings to different ethnicities, illness severities, genders (of both patients and treating physicians), and physician speciality is an important avenue for future research. We also used a cross-sectional study design, we relied upon patient self-reported outcomes, and participants were asked to recall physician behaviours in retrospect. As such, claims regarding causality cannot be made, recall bias may be present, and future research should supplement outcome measures with objective medical records. Additionally, although SDT suggests that individuals who are more autonomously motivated are more likely to engage in behaviours towards growth and psychological well-being, there are exceptions whereby some individuals engage in passive or counterproductive behaviours that ultimately impact growth, internalisation, and well-being. In line with this, we acknowledge that individual differences in patient factors may thwart physicians’ best efforts to develop strong physician–patient relationships and to foster satisfaction of basic psychological needs and autonomous motivation. It was beyond the scope of the current study to investigate the relative importance of each of the three basic psychological needs (i.e. autonomy, competence relatedness) and model their unique relationship with motivation and patient outcomes. This would be an important direction for future research to allow time-poor physicians to tailor consultations to best suit patient needs. Importantly, broader healthcare system factors such as a physician’s available consultation time and capacity for continuity of care may influence a physician’s ability to address a patient’s need for autonomy, competence, and relatedness. Future research could quantify this and discuss ways to address these systemic barriers.

## Conclusion

In summary, our study has provided a new model of the relationships between the physician–patient relations, basic psychological needs, motivation, and patient outcomes in the context of chronic disease. Stronger physician–patient relationships were shown to indirectly predict better physical and mental health, as well as greater adherence to treatment. This occurred through serial mediation, whereby stronger physician–patient relationships predicted need satisfaction, which in turn predicted autonomous motivation. In contrast, weaker physician–patient relationships indirectly predicted poorer mental health and lower adherence to treatment. The physician–patient relationship was also directly associated with controlled motivation and indirectly associated with autonomous motivation, through need satisfaction. Together, the findings suggest that physicians and policy makers should consider the important role of satisfaction of basic psychological needs when aiming to motivate patients to initiate and maintain health behaviours. Findings also confirm the negative consequences of frustration of psychological needs and controlled motivation for physical and mental health.

## Data Availability

The data is not available to protect the privacy of participants.
